# Awareness of Changes in E-cigarette Regulations and Behavior Before and After Implementation: A Longitudinal Survey of Smokers, Ex-smokers, and Vapers in the United Kingdom

**DOI:** 10.1093/ntr/ntz008

**Published:** 2019-01-25

**Authors:** Hyun S Lee, Samara Wilson, Timea Partos, Ann McNeill, Leonie S Brose

**Affiliations:** 1 GKT School of Medical Education, King’s College London, London, UK; 2 Addictions, Institute of Psychiatry, Psychology and Neuroscience, King’s College London, London, UK

## Abstract

**Introduction:**

In line with the European Union’s Tobacco Products Directive (TPD), new regulations for electronic cigarettes implemented in the United Kingdom between May 2016 and May 2017 included limiting refills to 10 mL, tank and cartridge sizes to 2 mL, and nicotine concentrations to 20 mg/mL.

**Aims:**

To investigate the (1) awareness of new regulations, (2) product use before and after implementation, and (3) association between use of compliant products and subsequent smoking.

**Methods:**

A UK online longitudinal survey of smokers, ex-smokers, and vapers was conducted between May and June 2016 (wave 4) and September 2017 (wave 5).The following methods were used: (1) to assess awareness of changes, proportions were calculated by smoking and vaping status (*n* = 1606). (2) Comparison of refill volume, tank and cartridge volumes, nicotine concentration at waves 4 and 5 (*n* = 199–388) was conducted. (3) Association was studied between number of TPD-compliant products used at wave 4 and smoking at wave 5, adjusted for wave 4 vaping status, age, gender, income, urges to smoke, and device type (*n* = 480).

**Results:**

Awareness of regulations was highest for refill volume (10.1%; 37.4% among exclusive vapers) and nicotine concentration (9.5%; 27.3%). Higher proportions used TPD-compliant refill volumes (60.0%–73.7%, χ^2^(1) = 10.9, *p* = .001) and nicotine concentrations (89.2%–93.9%, χ^2^(1) = 7.41, *p* = .007) in wave 5 than wave 4, with little change for tank or cartridge volumes (77.1–75.5%, χ^2^(1) = 0.38, *p* = .540). The likelihood of smoking was similar for those using no or one TPD-compliant products as it was for those using two (OR = 1.10, 95% CI = 0.47–2.59) or three (OR = 1.56, 95% CI = 0.69–3.55).

**Conclusion:**

Several months after full implementation, awareness of new regulations was low and most vapers used TPD-compliant products. Use of compliant products was not associated with subsequent smoking.

**Implications:**

Using a longitudinal survey at the beginning and a few months after the end of the transition period for implementation of new regulation on electronic cigarettes, this is the first study to assess awareness of regulation and use of compliant products. After full implementation, awareness of changes was low overall (smokers, ex-smokers, and vapers combined) although higher among those who vaped. Nevertheless, most vapers (74%–94%) used products that were compliant with the new regulations and the use of products compliant with incoming regulations did not predict whether they were smoking cigarettes after implementation.

## Introduction

An electronic cigarette (e-cigarette) or vaping device consists of a power source, heater, and a liquid which is aerosolized to deliver varying concentrations of nicotine to the user (vaper). Since their commercial introduction in the mid-2000s, they have become increasingly popular, especially as a smoking cessation tool.^1,2^ Recent reports from both the US National Academies of Science, Engineering, and Medicine, and Public Health England state that e-cigarettes are less harmful than smoking.^3,4^ However, there is much debate surrounding e-cigarette regulation and current regulatory approaches vary substantially around the world.^5^ As of October 2017, a total of 83 countries had enacted laws regarding e-cigarettes: 27 countries banned sales of all types of e-cigarettes and nine countries banned the sale of nicotine-containing e-cigarettes.^6^ It is not yet clear which regulatory approach is of the greatest benefit to public health.

In 2014, the European Parliament formally approved a revision of the 2001 Tobacco Products Directive (TPD) after an extensive consultation period, including lobbying campaigns by the tobacco industry.^7,8^ It became fully applicable in European Union Member States on May 20, 2016, although Member States may have differed in how they translated the TPD to national legislation, and for some clauses Member States had until May 2017 for full implementation. The revised European Union TPD introduced regulations specific to e-cigarettes. Cartridges and refillable tanks were now limited to a maximum volume of 2 mL. The refill liquids (e-liquids) were limited to a maximum volume of 10 mL per container sold, with maximum nicotine concentrations of 20 mg/mL. In addition, stricter requirements on ingredients for liquids, increased regulation of promotional materials, and requirements for packaging and warning labels were also introduced.^9^

In the United Kingdom, the Directive was translated into law via the “Tobacco and Related Products Regulations 2016.”^10^ Manufacturers and importers of e-cigarettes or refill containers were required, by November 20, 2016, to notify the Medicines and Healthcare products Regulatory Agency (MHRA), for all products already or to be marketed before May 19, 2016. A 1-year transitional period for noncompliant products on the market ended on May 20, 2017, from which all products were required to be fully compliant with the TPD. For products containing more than the TPD-stipulated volumes and concentrations, manufacturers may apply for a medicinal license; no licensed products were commercially available as of July 2018. Using the terminology of the behavior change wheel,^11^ legislation is one way of changing behavior. In this case, a restriction of the availability of products leads to reduced opportunity to purchase specific devices or concentrations of nicotine, leading to a change in vapers’ behavior. According to surveys conducted in March 2016, of the 95% of vapers who were using nicotine, 9% reported using nicotine concentrations greater than those permitted under the TPD (20 mg/mL) and an additional 12% were not sure.^12^ As such, there had been concerns that limitation on the strength of e-liquids and size of refills may make it more likely for vapers to return to tobacco products.^13^

To date, there have been no published studies investigating if those potentially affected by the changes in the regulations were aware of the changes or whether they have changed their behavior following implementation of the European Union TPD. Therefore, the first aim of this study was to investigate awareness of the new regulations among smokers, ex-smokers, and vapers. The second aim was to investigate all current and former vapers’ product use before and after full implementation of the TPD. The third aim was to investigate among vapers whether using non TPD-compliant products before implementation was associated with tobacco smoking after implementation.

## Methods

### Study Design and Sample

This study was a subgroup analysis of data from a longitudinal online survey of smokers, ex-smokers, and vapers from the United Kingdom. Members of an online panel managed by Ipsos MORI were invited to complete surveys. For each completed survey, participants receive points that may be exchanged for shopping vouchers or used to enter prize draws. Through initial screening in 2012 (*n* = 23 785), 6165 were found to be past-year smokers, making them eligible for the study. Quotas on age, gender, and UK region were imposed at the recruitment stage, which amounted to 5000 participants completing the survey in the first wave (December 2012). Wave 2 (2013) and wave 3 (2014) followed up with 2182 and 1519 participants, respectively. Wave 4 was conducted in May and June 2016 just as the TPD was introduced in the United Kingdom. In addition to those followed up from previous waves (*n* = 931), 2403 participants were newly recruited, with quotas to ensure the overall sample retained broad representativeness of the United Kingdom by sex, age, and region. Current vapers who had never smoked were also eligible for this wave. Another follow-up wave (wave 5) conducted in September 2017, (4 months after the final implementation deadline of May 2017) was completed by 1720 participants.

Participants with contradictory responses regarding their e-cigarette use history (eg, those who stated having never vaped in wave 5, but reported using e-cigarettes in wave 4) were excluded from further analysis, resulting in 1606 participants in the final analysis (representing 48.1% of wave 4 and 93.3% of wave 5). Of these, 682 (42.5% of *n* = 1606) participants reported never having used e-cigarettes so were not asked any questions about their use or purchasing patterns.

### Measures

**Figure 1.**
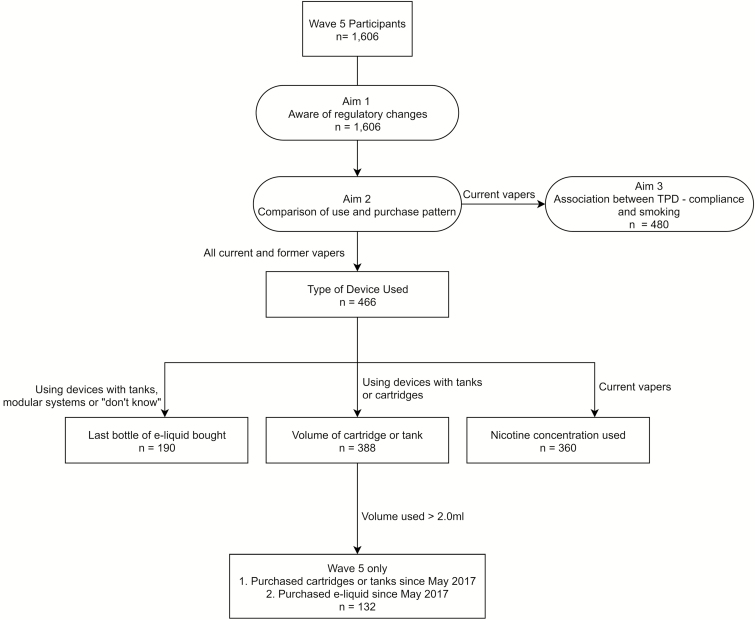
Flowchart of question routing used.

Demographics assessed in wave 4 included age (continuous), gender (male, female), income (collapsed into ≤£15 000; £15 001–£30 000; >£30 000; don’t know or prefer not to say). Box 1 presents measures related to smoking and vaping. Current smoking and current vaping status were combined to classify respondents into the following categories: Exclusive vapers, Dual users (smoking and vaping), Exclusive smokers, and Neither. All respondents were asked about their awareness of each item listed in the question about changes in e-cigarette regulations. The remainder of the survey used routing to ask questions depending on answers to previous items to ensure respondents were only asked questions applicable to them. As such, each item had varying number of responses (Figure 1). In brief, all current and former vapers were asked to choose the device they used most; those who used or had used refillable e-cigarette devices were asked about the volume of e-liquid refills bought; those who used or had used cartridge or tank devices were also asked about the volume of cartridge or tank used. All current vapers were asked about the concentration of nicotine used. If multiple responses were chosen, they were asked to state the concentration used most often and this value was used for analysis. Volumes and concentrations used or bought were then used to classify products used as being compliant with the limits set in the TPD or noncompliant. Finally, all current and recent past smokers (those who had quit after the preceding wave) were asked about their strength of urges to smoke.

**Box 1. Measures related to smoking and vaping**

Smoking status

Could you please tell us which of the following best applies to you now?

a) I smoke cigarettes (including hand-rolled) every day^1^
b) I smoke cigarettes (including hand-rolled), but not every day^1^
c) I do not smoke cigarettes at all, but I do smoke tobacco of some kind (e.g. pipe or cigar)^1^
d) I have stopped smoking completely in the last year (i.e. since May 2015)/since the last survey in December 2014) [In Wave 4, different time spans for previous participants and new recruits; in Wave 5, May/June 2016 for all participants]
e) I stopped smoking completely more than a year ago (i.e. before May 2015)/before the last survey in December 2014 [In Wave 4, different time spans for previous participants and new recruits; in Wave 5, May/June 2016 for all participants]
f) I have never been a smoker

^1^ Categorised as current smoker

Vaping status

Could you please tell us which of the following best applies to you now?

a) I currently vape/use e-cigarettes daily^1^
b) I currently vape/use e-cigarettes but not every day^1^
c) I have tried vaping/an e-cigarette once or a few times
d) I stopped vaping/using e-cigarettes since the in the last year [In wave 5, May/June 2016]
e) I stopped vaping/using e-cigarettes over a year ago [In wave 5, May/June 2016]
f) I have never vaped/used e-cigarettes.

^1^ Categorised as current vaper

Awareness of changes in e-cigarette regulation

The UK has recently implemented changes to the law relating to electronic cigarettes, under the new Tobacco Products Directive (TPD). Have you been aware of any changes to…

- Requirement to notify the government of ingredients and harmful effects
- Requirements relating to quality control
- The size of the e-liquid refill containers
- The size of pre-filled cartridges
- The nicotine strength of e-liquids
- Additives in e-liquids
- Packaging safety standards
- The information provided with e-cigarettes and e-liquids
- Warning labels on packaging
- Rules about sales of e-cigarettes to minors^1^
- Locations where e-cigarettes may be sold^2^
- Packaging colours^2^
- Other
- Not aware of any changes

^1^Not part of the TPD-related changes, but also changed in 2016/17 in the UK; ^2^Incorrect response

Device

What electronic cigarette or vaping device do you currently use/did you use the most?

a) A disposable e-cigarette or vaping device^1^
b) An e-cigarette or vaping device that uses replaceable pre-filled cartridges^1^
c) An e-cigarette or vaping device with a tank that you refill with liquids^2^
d) A modular system that you refill with liquids^2^
e) Don’t know^1^

^1^Categorised as “cigalikes” (resembling tobacco cigarettes); ^2^Categorised as refillable tank devices.

Volume of e-liquid refills

What size was the last bottle of e-liquid you bought?

a) Please give your answer in ml
b) Don’t know/couldn’t say [not provided in wave 5]

Volume of cartridge or tank

What is the volume/capacity of the cartridge(s) or tanks you usually use/used with your e-cigarette/vaping device?

a) Less than 1.0 ml^1^
b) 1.0–1.5 ml^1^
c) 1.6–2.0 ml^1^
d) 2.1–3.0 ml
e) 3.1–4.0 ml
f) More than 4.0 ml
g) Don’t know

^1^Compliant with TPD.

Concentration of nicotine

What strengths of nicotine do you use when vaping/using your e-cigarette?

a) No nicotine^1^
b) 1–8 mg/ml^1^
c) 9–14 mg/ml^1^
d) 15–20 mg/ml^1^
e) 21–24 mg/ml
f) 25 mg/ml
g) Don’t know

^1^Compliant with TPD

Urges to smoke

How much of the time have you felt the urge to smoke in the past 24 hours?

a) Not at all^1^
b) A little of the time
c) Some of the time
d) A lot of the time
e) Almost all of the time
f) All the time
g) Don’t know

[If b–f]: In general, how strong have the urges to smoke been?

a) Slight^1^
b) Moderate^2^
c) Strong^3^
d) Very Strong^3^
e) Extremely Strong^3^
f) Don’t know

^1^Low; ^2^Moderate; ^3^Strong

### Analysis

To assess awareness of changes in e-cigarette regulations (aim 1), proportions were calculated for each area of TPD and non-TPD regulatory changes by vaping and smoking status.

To compare product usage between the two waves (aim 2), McNemar’s tests and McNemar–Bowker test of symmetry were used. Only those who had answered identical questions in both surveys were analyzed. To maximize sample sizes, observations with missing values were deleted separately for each question at analysis.

For aim 3, a bivariate logistic regression assessed the relationship between the number of TPD-compliant products used or purchased (volume of refills, volume of cartridge or tank, concentration of nicotine) at wave 4 and being a smoker (exclusively or as part of dual use) at wave 5. Multivariable logistic regressions adjusted for participants’ vaping status, age, gender, income, strength of urges to smoke at wave 4, and type of device used. Six respondents who stated “don’t know” for device type were combined with those using cigalike devices for this analysis. Respondents were only asked about TPD categories applicable to them, that is, someone using a cigalike type e-cigarette (which are not refillable) was not asked about the volume of refills. Participants who used disposable devices were not asked about volume of refills or tanks/cartridges. To maximize sample size, such respondents and those who responded “don’t know” were treated as using TPD-compliant products. However, to ensure the validity of results, we also conducted sensitivity analyses with the “don’t know” responses being treated as noncompliant for both aims 2 (product use) and 3 (association with smoking at follow-up). For aim 3, a further sensitivity analysis was conducted with “don’t know” and unknown responses excluded.

SAS v. 9.4M4 (SAS Institute, Cary, NC) and SPSS v. 24 (IBM Corp, New York, NY) were used for analysis.

## Results

A summary of baseline characteristics is presented in Table 1.

**Table 1.** Baseline Characteristic of Sample at Wave 4

	All participants (*n* = 1606)
Age	
Mean (SD)	49.7 (14.9)
Gender, *n* (%)	
Male	903 (56.2)
Income, *n* (%)	
Less than £15 000	331 (20.6)
£15 001–£30 000	491 (30.6)
More than £30 000	632 (39.4)
Unknown	152 (9.5)
Tobacco use, *n* (%)	
Daily	919 (57.2)
Nondaily	176 (11.0)
Other tobacco	82 (5.1)
Ex-smoker	419 (26.1)
Never smoked	10 (0.6)
E-cigarette use, *n* (%)	
Daily	281 (17.5)
Nondaily	199 (12.4)
Tried	310 (19.3)
Ex-vaper	134 (8.3)
Never vaped	682 (42.5)

### Awareness of Changes in Regulation

Although over a quarter of respondents responded that they were aware of changes, only 10.1% of participants were aware of changes to the size of e-liquid refill containers and 9.5% of changes to regulations for the nicotine strength of e-liquids. All other changes were known to fewer than 8% of participants, including the three non-TPD regulatory items that were endorsed by about 4% of participants (Table 2). Among exclusive vapers and dual users, awareness was above 10% for sizes of e-liquid refills (exclusive vapers: 37.4%; dual users: 13.2%), nicotine strengths (exclusive vapers: 27.3%; dual users: 15.1%), size of prefilled cartridges (exclusive vapers: 17.7%; dual users: 10.7%), and warning labels on packaging (exclusive vapers: 17.2%; dual users: 14.2%; Table 2).

**Table 2.** Awareness of Regulation by Wave 5 Vaping and Smoking Status

	Wave 5				
	Exclusive vaper, *n* = 198	Dual user, *n* = 318	Exclusive smoker, *n* = 783	Neither^a^, *n* = 307	Total, *N* = 1606
Aware of any regulatory changes, %	58.6	49.9	13.8	15.0	26.6
Aware of specific changes, %					
The size of e-liquid refill containers	37.4	13.2	4.3	3.9	10.1
The nicotine strength of e-liquids	27.3	15.1	4.2	5.9	9.5
Warning labels on packaging	17.2	14.2	4.1	4.6	7.8
The size of prefilled cartridges	17.7	10.7	2.7	2.0	5.9
Rules about sales of e-cigarettes to minors^b^	9.1	8.2	2.0	2.3	4.2
Packaging colors^c^	3.5	7.2	3.2	2.9	4.0
Packaging safety standards	9.1	6.9	1.5	2.0	3.6
Locations where e-cigarettes may be sold^c^	3.5	7.2	2.2	3.3	3.6
Additives in e-liquids	5.6	6.9	1.8	1.3	3.2
The information provided with e-cigarettes and e-liquids	7.6	6.9	1.1	1.3	3.1
Requirements relating to quality control	8.1	6.3	1.1	1.0	3.0
Requirement to notify the government of ingredients and harmful effects	4.0	4.4	1.7	0.3	2.2

^a^Ex-smokers who do not vape.

^b^Not part of the Tobacco Products Directive-related changes, but also changed in 2016/2017 in the United Kingdom.

^c^Incorrect response, this aspect did not change.

### Change in Product Use

A comparison of e-cigarette use and purchasing behaviors between wave 4 and wave 5 is shown in Table 3. Wave 5 had a statistically greater proportion of current vapers compared to wave 4. More respondents reported using devices with refillable tanks as their main device in wave 5 (χ2 = 14.0, *p* = .002).

**Table 3.** Comparison of Wave 4 versus Wave 5 E-cigarette Use

	Wave 4	Wave 5	McNemar
Current e-cigarette use (*N* = 1606)			χ2(1) = 4.7, *p* = .030
Yes	480 (29.9)	516 (32.1)	
No	1126 (70.1)	1090 (67.9)	
Type of device used^a^ (*n* = 466)			χ2(3) = 14.0, *p* = .002
Don’t know	14 (3.0)	18 (3.9)	
Cigalike	170 (36.5)	138 (29.6)	
Refillable tanks	282 (60.5)	310 (66.5)	
Volume of e-liquid refill bought^b^ (*n* = 190)			χ2(1) = 10.9, *p* = .001
TPD compliant (≤10 mL)	95 (50.0)	140 (73.7)	
Noncompliant with TPD (>10 mL)	76 (40.0)	50 (26.3)	
Don’t know	19 (10.0)	0 (0)	
*If noncompliant, purchased e-liquid refills since May 2017* (*n*, %)			
Yes		15 (30.0)	
No		8 (16.0)	
Unknown		27 (54.0)	
Volume cartridge/tank used^b^ (*n* = 388)			χ2(1) = 0.38, *p* = .540
TPD compliant (≤2 mL)	139 (35.8)	149 (38.4)	
Noncompliant with TPD (>2 mL)	89 (22.9)	95 (24.5)	
Don’t know	160 (41.2)	144 (37.1)	
*If noncompliant, purchased cartridges or tanks since May 2017* (*n*, %)			
Yes		48 (50.5)	
No		46 (48.4)	
Don’t know		1 (1.1)	
Nicotine concentration used^b^ (*n* = 360)			χ2(1) = 7.41, *p* = .007
TPD compliant (<2%)	285 (79.2)	305 (84.7)	
Noncompliant with TPD	39 (10.8)	22 (6.1)	
Don’t know	36 (10.0)	33 (9.2)	
Changed brand of e-cigarette or vaping device (*n* = 440)			
Yes		52 (11.8)	
No		371 (84.3)	
Don’t know		17 (3.9)	

TPD = Tobacco Products Directive.

^a^McNemar–Bowker’s test of symmetry was used for analysis.

^b^“Don’t know” responses were treated as being TPD-compliant for analysis.

Among vapers who reported using devices with refillable tanks in both waves (*n* = 190), a higher proportion of users purchased TPD-compliant refill volumes at wave 5. Of those who reported purchasing non-TPD-compliant volumes at wave 5 (*n* = 50), 30% reported purchasing refills after the implementation deadline of May 2017 (Table 3). A significantly greater proportion of respondents reported using TPD-compliant nicotine concentrations at wave 5 than in wave 4 (93.9% vs. 89.2%, Table 3). The difference in volumes purchased and nicotine concentrations used remained significant in the sensitivity analyses where “don’t know” values were treated as noncompliant (Supplementary Table 1).

No significant differences were seen in the proportion using TPD-compliant volumes for cartridges or tanks in their e-cigarette devices before and after TPD implementation. Among those using cartridges or tanks that exceeded TPD-compliant volumes, 50.5% of respondents had purchased them after May 2017 (Table 3). No significant differences between waves were observed in the sensitivity analysis of volumes used where “don’t know” observations were treated as being noncompliant (Supplementary Table 1).

### Association Between Use of TPD-Compliant Products at Wave 4 and Tobacco Smoking at Wave 5

There were 480 participants who at wave 4 were exclusively vaping (*n* = 182, 37.9%) or vaping and smoking (dual use, *n* = 298, 62.1%). At wave 5, 198 (41.2%) of them were vaping and smoking, 162 (33.8%) exclusively vaping, 75 (15.6%) exclusively smoking, and 45 (9.4%) had stopped both. In bivariate analyses, using products compliant with all three TPD regulations was associated with 2.72 times greater odds of being a smoker (95% CI = 1.52–4.85) when “don’t know” responses were treated as using compliant products (Table 4). The association was no longer significant once adjusted for vaping status, age, gender, income, device type, and strength of urges to smoke at wave 4. The sensitivity analyses where “don’t know” responses were treated as using noncompliant products similarly showed no significant association between use of compliant products and subsequent smoking once adjusted (Supplementary Table 2). Further analysis where all unknown responses were excluded also showed no significant association between the use of compliant products and smoking at wave 5 (Supplementary Table 3).

**Table 4.** Bivariate and Multivariable Associations With Smoking (Dual Use or Exclusively Smoking) at Wave 5

Wave 4 characteristic		*n*	Unadjusted OR (95% CI)	*p*	Adjusted^a^ OR (95% CI)	*p*
Number of TPD-compliant behaviors^b^	0 or 1	57	1		1	
	2	125	1.24 (0.66–2.34)	.51	1.10 (0.47–2.58)	.83
	3	298	2.72 (1.52–4.85)	<.001	1.58 (0.70–3.59)	.27
Vaping status	Exclusive vaper	182	1		1	
	Dual user	298	23.83 (14.54–39.08)	<.001	11.60 (6.08–22.14)	<.001
Age		480	0.98 (0.97–0.99)	.005	0.99 (0.97–1.01)	.28
Gender	Male	286	1		1	
	Female	194	0.95 (0.66–1.38)	.80	1.10 (0.66–1.82)	.72
Income	≤£15 000	88	1		1	
	£15 001–£30 000	133	1.04 (0.61–1.80)	.87	1.00 (0.48–2.10)	1.00
	>£30 000	218	1.10 (0.67–1.82)	.71	0.95 (0.49–1.87)	.89
	Unknown	41	0.54 (0.25–1.14)	.11	0.96 (0.34–2.71)	.94
Device type	Cigalike	183	1		1	
	Refillable	297	0.48 (0.33–0.70)	<.001	0.71 (0.41–1.25)	.24
Strength of urges to smoke	Low	87	1		1	
	Moderate	172	4.15 (2.40–7.16)	<.001	1.25 (0.61–2.59)	.54
	Strong	130	6.88 (3.71–12.77)	<.001	1.48 (0.65–3.35)	.35
	Unknown	91	0.15 (0.06–0.35)	<.001	0.30 (0.12–0.77)	.01

CI = confidence interval; OR = odds ratio.

^a^Adjusted for wave 4 vaping status, age, gender, income, device type and strength of urges to smoke.

^b^“Don’t know” treated as being Tobacco Products Directive-compliant.

## Discussion

In this subgroup analysis of a longitudinal survey of smokers and ex-smokers in Great Britain, several months after the end of the transition period for implementation, awareness of the new regulations was very low overall with less than 10% of participants reporting awareness for most of the regulations, although higher for those who vaped. Limitations to the size of refill bottles and nicotine strength were most frequently correctly identified, whereas even vapers were rarely aware of other changes. This may suggest that there had been little noticeable change. The higher rates of awareness of changes reported by vapers compared to nonusers are similar to those seen with smokers and TPD-induced packaging changes.^14^ There had been an increase in the use of TPD-compliant products from before to after full implementation of the TPD with fewer vapers reporting purchasing noncompliant refill or tank volumes or using nicotine concentrations above those permitted under the TPD. Neither the main nor sensitivity analyses indicated associations between the use of TPD-compliant products at baseline and smoking at follow-up when adjusting for baseline characteristics.

The purchasing behavior of users is largely constrained by context; the availability of products influences purchasing choices, in that users do not necessarily make a conscious choice to purchase compliant products if this is all that is available. In this context, awareness of regulation is not a prerequisite for a change in behavior. Additional changes to the context will have influenced behavior. For example, other surveys suggested low proportions of vapers using high-strength nicotine before implementation of new legislation.^15^ Even before the implementation of the TPD, many common and popular volumes and concentrations were already in line with the incoming regulations.^16^ One reason behind this could be that newer devices are more efficient at nicotine delivery, requiring less nicotine content to achieve the same effect.^17^ Many products being in line with legislation ahead of time can also partly explain low awareness of changes to legislation as there would have been little noticeable change for many users.

In the newly restricted context, some vapers achieve flexibility by buying large bottles of nicotine-free liquids (not subject to TPD regulations) and adding small nicotine “shots”^4^; this enables them to achieve the desired strength and in a larger quantity while purchasing only TPD-compliant products. The present data were not able to assess the extent of these behaviors however. More respondents used refillable tanks, which is in line with a transition to more advanced, and potentially more effective, models over time.^18^ Finally, although new devices needed to comply with the new regulations, devices sold prior to implementation of the revised European Union TPD may continue to be used if they were still usable.

We assumed that those using high nicotine strengths or large volumes before implementation of TPD regulation were more susceptible to smoking post-implementation based on the assumption that higher nicotine content or volume vaped was at least to a degree used in accordance with participants’ dependence. Like more conventional nicotine-replacement therapies, e-cigarette nicotine concentrations can be self-titrated to the user’s needs. Studies have shown self-directed use of higher nicotine concentrations or increased number of puffs by vapers to reduce craving for traditional tobacco products.^19,20^ In this study, however, those who used products subsequently restricted by the TPD were no more likely to smoke at follow-up than those who used products that were not then restricted. It is worth noting that the United Kingdom has a very high level of effective tobacco control policies^21^, and associations may be different in the context of other countries and regulatory frameworks.

Findings warrant replication for confirmation as there are several limitations to our study. Limitations that restrict wider interpretations include the sample population being sampled to represent the general population, which may not reflect the different demographics of smokers and vapers. Substantial loss to follow-up further reduced the representativeness of the findings. Owing to the conditional routing used in the online questionnaire, the number of observations present in both waves are small for some items asked. Only those using modular devices with tanks were asked all three TPD-related questions. Cigalike device users were not asked about refill sizes purchased, and disposable device users were not asked about refills or volume of the device. We treated such products as being compliant in our study as such devices are very unlikely to be larger than permitted and no refills (compliant or not) were necessary. As such, respondents who used products that were compliant with all three TPD regulations are likely to have been nonmodular device users in our analysis. A related limitation was participants not knowing the details of the products used. Through sensitivity and multivariable analyses, we have attempted to minimize the above limitations as much as possible. Furthermore, in general, the veracity of self-reported responses cannot be ascertained in surveys. We have attempted to rectify this by removing inconsistent data, while keeping the largest sample size possible to increase power and reduce the impact of such observations.

Despite limitations, this study has several strengths. To our knowledge, this is the first study to use data collected during the early TPD implementation and post-implementation to allow a direct comparison. The sample was selected from the general population of the United Kingdom via a large survey service to limit selection bias and the variety of items asked in the survey allows for better adjustment in analysis. By investigating the early time frame following TPD implementation, this study provides a referential base for future analyses, after a longer period of implementation.

## Conclusion

Several months after full implementation of new regulations (European TPD) for e-cigarettes, awareness of changes was very low albeit higher among those who vaped. After implementation of regulation, most but not all vapers were using products compliant with new restrictions on nicotine concentrations in e-liquid and volumes of e-liquid refills purchased. Among baseline vapers, there was no association between smoking at follow-up, and whether devices, e-liquids, and refills used before implementation were in line with the new regulations.

## Funding

Cancer Research UK (C52999/A21496; C57277/A23884) funded the work reported in this manuscript. LB’s post is funded by a CRUK/BUPA Foundation Cancer Prevention Fellowship (C52999/A19748). LB and AMcN are part of the UK Centre for Tobacco and Alcohol Studies, a UK Clinical Research Collaboration Public Health Research: Centre of Excellence. Funding from the Medical Research Council, British Heart Foundation, Cancer Research UK, Economic and Social Research Council, and the National Institute for Health Research under the auspices of the UK Clinical Research Collaboration is gratefully acknowledged (MR/K023195/1).

## Declaration of Interest

*The authors declare no competing interests.*

## Supplementary Material

ntz008_suppl_Supplementary_Material

## References

[1] Noel JK, Rees VW, Connolly GN Electronic cigarettes: a new “tobacco” industry? Tob Control. 2011;20(1):81. 20930060 10.1136/tc.2010.038562

[2] Kaisar MA, Prasad S, Liles T, Cucullo L A decade of e-cigarettes: limited research & unresolved safety concerns. Toxicology. 2016;365:67–75. 27477296 10.1016/j.tox.2016.07.020 PMC4993660

[3] National Academies of Sciences E, Medicine. Public Health Consequences of E-cigarettes. Washington, DC: National Academies Press; 2018. 29894118

[4] McNeill A, Brose LS, Calder R, Bauld L, Robson D. Evidence Review of E-cigarettes and Heated Tobacco Products 2018. A Report Commissioned by Public Health England. London, UK: Public Health England; 2018.

[5] Kennedy RD, Awopegba A, De León E, Cohen JE Global approaches to regulating electronic cigarettes. Tob Control. 2017;26(4):440–445. 27903958 10.1136/tobaccocontrol-2016-053179 PMC5520254

[6] Institute for Global Tobacco Control. *Country Laws Regulating E-cigarettes: A Policy Scan* 2017 http://globaltobaccocontrol.org/e-cigarette/country-laws-regulating-e-cigarettes. Accessed February 18, 2018. PMC1763895 9825415

[7] Hiilamo H, Glantz SA Old wine in new bottles: tobacco industry’s submission to European Commission tobacco product directive public consultation. Health Policy. 2015;119(1):57–65. 25467283 10.1016/j.healthpol.2014.11.002 PMC4453831

[8] Peeters S, Costa H, Stuckler D, McKee M, Gilmore AB The revision of the 2014 European tobacco products directive: an analysis of the tobacco industry’s attempts to “break the health silo.” Tob Control. 2016;25(1):108–117. 25713313 10.1136/tobaccocontrol-2014-051919 PMC4669229

[9] European Commission. Tobacco Products Directive. 2014 https://ec.europa.eu/health/sites/health/files/tobacco/docs/dir_201440_en.pdf Accessed February 1, 2018.

[10] United Kingdom Government. The Tobacco and Related Products Regulations. 2016 http://www.legislation.gov.uk/uksi/2016/507/contents/made Accessed February1, 2018.

[11] Michie S, van Stralen MM, West R The behaviour change wheel: a new method for characterising and designing behaviour change interventions. Implement Sci. 2011;6(1):42. 21513547 10.1186/1748-5908-6-42 PMC3096582

[12] Action on Smoking and Health. *Use of Electronic Cigarettes (Vapourisers) Among Adults in Great Britain 2016* 2016 http://www.ash.org.uk/files/documents/ASH_891.pdf. Accessed February 18, 2018.

[13] Rough E, Barber S *Briefing Paper: The Regulation of E*-*cigarettes*. London, UK: House of Commons Library; 2017.

[14] Bogdanovica I, Opazo Breton M, Langley T, Britton J Awareness of standardised tobacco packaging among adults and young people during the final phase of policy implementation in Great Britain. Int J Environ Res Public Health. 2017;14(8):858. 10.3390/ijerph14080858 PMC5580562 28788103

[15] Action on Smoking and Health. *Use of E-cigarettes (Vapourisers) Among Adults in Great Britain 2018* 2018 http://ash.org.uk/download/use-of-e-cigarettes-among-adults-in-great-britain-2017/. Accessed October 1, 2018.

[16] Etter JF, Zäther E, Svensson S Analysis of refill liquids for electronic cigarettes. Addiction. 2013;108(9):1671–1679. 23701634 10.1111/add.12235

[17] Farsalinos KE, Spyrou A, Tsimopoulou K, Stefopoulos C, Romagna G, Voudris V Nicotine absorption from electronic cigarette use: comparison between first and new-generation devices. Sci Rep. 2014;4:4133. 24569565 10.1038/srep04133 PMC3935206

[18] Hitchman SC, Brose LS, Brown J, Robson D, McNeill A Associations between e-cigarette type, frequency of use, and quitting smoking: findings from a longitudinal online panel survey in Great Britain. Nicotine Tob Res. 2015;17(10):1187–1194. 25896067 10.1093/ntr/ntv078 PMC4580313

[19] Dawkins LE, Kimber CF, Doig M, Feyerabend C, Corcoran O Self-titration by experienced e-cigarette users: blood nicotine delivery and subjective effects. Psychopharmacology (Berl). 2016;233(15-16):2933–2941. 27235016 10.1007/s00213-016-4338-2

[20] Farsalinos KE, Romagna G, Tsiapras D, Kyrzopoulos S, Voudris V Evaluating nicotine levels selection and patterns of electronic cigarette use in a group of “vapers” who had achieved complete substitution of smoking. Subst Abuse. 2013;7:139–146. 24049448 10.4137/SART.S12756 PMC3772898

[21] Luk Joossens MR *The Tobacco Control Scale* 2016 https://www.tobaccocontrolscale.org/. Accessed October 18, 2018.

