# Supplementary material for: Awareness of Changes in E-cigarette Regulations and Behavior Before and After Implementation: A Longitudinal Survey of Smokers, Ex-smokers, and Vapers in the United Kingdom
Source: Nicotine Tob Res. 2019 Jan 25;22(5):705–12. doi: 10.1093/ntr/ntz008 (PMC7171274; doi:10.1093/ntr/ntz008)
Supplement: ntz008_suppl_Supplementary_Material [file ntz008_suppl_supplementary_material.docx]

**Lee, Wilson, Partos, McNeill & Brose, Awareness of changes in e-cigarette regulations and behaviour before and after implementation: A longitudinal survey of smokers, ex-smokers and vapers in the United Kingdom.**

**Supplementary Tables**

**Table S1: Comparison of Wave 4 vs Wave 5 e-cigarette use**

|  | **Wave 4** | **Wave5** | **Statistical tests^+^** |
| --- | --- | --- | --- |
| **Volume of e-liquid refill bought** (N=190) |  | |  |
| TPD compliant (≤10ml) | 95 (60.0) | 140 (73.7) | χ2 (1) =16.5, ***p*<0.001** |
| Non-compliant with TPD (>10ml) | 95 (40.0) | 50 (26.3) |  |
|  |  |  |  |
| **Volume cartridge/tank used** (n=388) |  | |  |
| TPD compliant (≤2ml) | 139 (35.8) | 149 (38.4) | χ2 (1) =0.8, *p* =0.369 |
| Non-compliant with TPD (>2ml) | 249 (64.2) | 239 (61.6) |  |
|  |  |  |  |
| **Nicotine concentration used** (n=360) |  |  |  |
| TPD compliant (<2%) | 285 (79.2) | 305 (84.7) | χ2 (1) =6.3, ***p* =0.012** |
| Non-compliant with TPD | 75 (20.8) | 55 (15.3) |  |

^+^ “Don’t know” responses were treated as being non-compliant for statistical comparisons

**Table S2: Bivariate and multivariate associations with smoking (dual use or exclusively smoking) at Wave 5. N=480**

| **Wave 4 characteristic** | **N** | **OR (95% CI)** | ***p*** | **Adjusted^+^ OR (95% CI)** | ***p*** |
| --- | --- | --- | --- | --- | --- |
| **TPD compliant behaviours** |  |  |  |  |  |
| 0 & 1 | 173 | 1 |  | 1 |  |
| 2 | 173 | 1.32 (0.87-2.02) | 0.20 | 1.19 (0.67-2.12) | 0.55 |
| 3 | 134 | 1.83 (1.15-2.91) | **0.01** | 1.30 (0.67-2.52) | 0.44 |
| **Vaping status** |  |  |  |  |  |
| Vaper | 182 | 1 |  | 1 |  |
| Dual-user | 298 | 23.83 (14.54-39.08) | **<0.001** | 12.21 (6.41-23.26) | **<0.001** |
| **Age** | 480 | 0.98 (0.97-0.99) | **0.005** | 0.99 (0.97-1.01) | 0.30 |
| **Gender** |  |  |  |  |  |
| Male | 286 | 1 |  | 1 |  |
| Female | 194 | 0.95 (0.66-1.38) | 0.80 | 1.12 (0.68-1.86) | 0.65 |
| **Income** |  |  |  |  |  |
| ≤£15,000 | 88 | 1 |  | 1 |  |
| £15,001-£30,000 | 133 | 1.04 (0.61-1.80) | 0.87 | 1.04 (0.50-2.18) | 0.91 |
| >£30,000 | 218 | 1.10 (0.67-1.82) | 0.71 | 0.95 (0.48-1.85) | 0.87 |
| Unknown | 41 | 0.54 (0.25-1.14) | 0.11 | 0.97 (0.35-2.74) | 0.96 |
| **Device Type** |  |  |  |  |  |
| Cigalike | 183 | 1 |  |  |  |
| Refillable | 297 | 0.48 (0.33-0.70) | **<0.001** | 0.66 (0.38-1.15) | 0.14 |
| **Strength of urges to smoke** |  |  |  |  |  |
| Low | 87 | 1 |  | 1 |  |
| Moderate | 172 | 4.15 (2.40-7.16) | **<0.001** | 1.19 (0.58-2.44) | 0.64 |
| Strong | 130 | 6.88 (3.71-12.77) | **<0.001** | 1.40 (0.62-3.16) | 0.41 |
| Unknown | 91 | 0.15 (0.06-0.35) | **<0.001** | 0.29 (0.11-0.74) | **0.009** |

Outcome modelled = smoking at wave 5

* “Don’t know” variables treated as being non-compliant

^+^ Adjusted for Wave 4 vaping status, age, gender, income, strength of urge to smoke

**Table S3: Bivariate and multivariate associations with smoking (as part of dual use or exclusively smoking) at Wave 5. N=157**

| **Wave 4 characteristic** | **N** | **OR (95% CI)** | ***p*** | **Adjusted^+^ OR (95% CI)** | ***p*** |
| --- | --- | --- | --- | --- | --- |
| **TPD compliant behaviours*** |  |  |  |  |  |
| 0 & 1 | 50 | 1 |  | 1 |  |
| 2 | 56 | 1.21 (0.56-2.62) | 0.63 | 1.21 (0.4-3.61) | 0.73 |
| 3 | 51 | 1.33 (0.61-2.93) | 0.47 | 1.89 (0.6-5.97) | 0.28 |
| **Vaping status** |  |  |  |  |  |
| Vaper | 86 | 1 |  | 1 |  |
| Dual-user | 71 | 17.68 (7.95-39.28) | **<0.001** | 6.68 (2.38-18.75) | **<0.001** |
| **Age** | 157 | 0.98 (0.96-1.00) | 0.20 | 0.97 (0.93-1.00) | 0.08 |
| **Gender** |  |  |  |  |  |
| Male | 103 | 1 |  | 1 |  |
| Female | 54 | 0.92 (0.47-1.79) | 0.80 | 1.1 (0.44-2.75) | 0.84 |
| **Income** |  |  |  |  |  |
| ≤£15,000 | 37 | 1 |  | 1 |  |
| £15,001-£30,000 | 31 | 0.49 (0.19-1.30) | 0.15 | 0.83 (0.21-3.32) | 0.80 |
| >£30,000 | 68 | 0.57 (0.24-1.35) | 0.20 | 0.53 (0.17-1.67) | 0.28 |
| Unknown | 21 | 0.29 (0.09-0.95) | **0.04** | 0.31 (0.06-1.57) | 0.16 |
| **Device Type** |  |  |  |  |  |
| Cigalike | 0 | - | - | - | - |
| Refillable | 157 | - | **-** | - | - |
| **Strength of urge to smoke** |  |  |  |  |  |
| Low | 42 | 1 |  | 1 |  |
| Moderate | 42 | 4.46 (1.78-11.15) | **<0.01** | 2.28 (0.73-7.18) | 0.16 |
| Strong | 30 | 8.00 (2.66-24.05) | **<0.001** | 3.00 (0.79-11.37) | 0.11 |
| Unknown | 43 | 0.10 (0.02-0.46) | **<0.01** | 0.16 (0.03-0.87) | **0.03** |

Outcome modelled = smoking at wave 5

* “Don’t know” and unknown responses excluded

^+^ Adjusted for Wave 4 vaping status, age, gender, income, strength of urges to smoke
